# Membrane transporters in a human genome-scale metabolic knowledgebase and their implications for disease

**DOI:** 10.3389/fphys.2014.00091

**Published:** 2014-03-11

**Authors:** Swagatika Sahoo, Maike K. Aurich, Jon J. Jonsson, Ines Thiele

**Affiliations:** ^1^Center for Systems Biology, University of IcelandReykjavik, Iceland; ^2^Molecular Systems Physiology Group, Luxembourg Centre for Systems Biomedicine, University of LuxembourgBelval, Luxembourg; ^3^Department of Biochemistry and Molecular Biology, Faculty of Medicine, University of IcelandReykjavik, Iceland; ^4^Department of Genetics and Molecular Medicine, Landspitali, National University Hospital of IcelandReykjavik, Iceland

**Keywords:** human metabolism, transport mechanisms, constraint-based modeling, inborn errors of metabolism, cancer, metabolic networks and pathways

## Abstract

Membrane transporters enable efficient cellular metabolism, aid in nutrient sensing, and have been associated with various diseases, such as obesity and cancer. Genome-scale metabolic network reconstructions capture genomic, physiological, and biochemical knowledge of a target organism, along with a detailed representation of the cellular metabolite transport mechanisms. Since the first reconstruction of human metabolism, Recon 1, published in 2007, progress has been made in the field of metabolite transport. Recently, we published an updated reconstruction, Recon 2, which significantly improved the metabolic coverage and functionality. Human metabolic reconstructions have been used to investigate the role of metabolism in disease and to predict biomarkers and drug targets. Given the importance of cellular transport systems in understanding human metabolism in health and disease, we analyzed the coverage of transport systems for various metabolite classes in Recon 2. We will review the current knowledge on transporters (i.e., their preferred substrates, transport mechanisms, metabolic relevance, and disease association for each metabolite class). We will assess missing coverage and propose modifications and additions through a transport module that is functional when combined with Recon 2. This information will be valuable for further refinements. These data will also provide starting points for further experiments by highlighting areas of incomplete knowledge. This review represents the first comprehensive overview of the transporters involved in central metabolism and their transport mechanisms, thus serving as a compendium of metabolite transporters specific for human metabolic reconstructions.

## Introduction

Membrane transporters mediate the transport of solutes across cell and organelle membranes. Transport processes can generate concentration gradients (e.g., active transport process) and membrane potentials (i.e., electrochemical gradient), and they contribute to the regulation of biochemical pathways by maintaining the cellular concentrations of substrates and products (e.g., GLUT proteins regulate the availability of glucose). Approximately 2000 genes in the human genome encode for transporters or transport-related proteins (Brunton et al., 2006). Defective metabolite transport processes have been associated with various pathological conditions, including inborn errors of metabolism (IEMs) (Camacho and Rioseco-Camacho, 1993; Kobayashi et al., 1993; Seow et al., 2004), obesity (Prudente et al., 2007), and cancer (Cooper et al., 2003; Macheda et al., 2005). Hence, knowledge of the cellular transport systems is fundamental to understanding human metabolism.

Genome-scale metabolic reconstructions (GENREs) integrate the genomic, physiological, and biochemical knowledge of a target organism (Palsson, 2006). GENREs are knowledge bases for metabolites, biochemical transformations/reactions, enzymes catalyzing the reactions, and genes that encode these enzymes. GENREs are available for more than 100 organisms, including human (Duarte et al., 2007; Thiele et al., 2013). GENREs can be easily converted into mathematical models and used for constraint-based modeling and analysis (COBRA), including flux balance analysis (Orth et al., 2010). Details on the procedures for GENRE and COBRA modeling are discussed elsewhere (Palsson, 2006; Thiele and Palsson, 2010a; Schellenberger et al., 2011). The comprehensive biochemical knowledge captured by GENREs includes gene-protein-reaction associations (GPRs), where individual metabolic/transport reactions are represented along with the genes that encode the enzyme/protein that catalyzes the reactions. These GPRs are Boolean relationships between the genes/transcripts and reactions with an “and” or “or” association. “And” indicates that the expression of all of the genes is necessary for the reaction(s) to be active (e.g., multi-enzyme complexes catalyzing a single reaction). An “or” relationship implies that any of the genes or gene products can catalyze the reaction (e.g., isozymes catalyzing the same reaction).

The first GENRE for human metabolism, Recon 1 (Duarte et al., 2007), captured the biochemical transformations occurring in cells in a stoichiometrically accurate manner. These reactions are distributed over seven intracellular compartments (i.e., cytoplasm, mitochondrion, Golgi apparatus, endoplasmic reticulum, lysosome, peroxisome, and nucleus). In addition, Recon 1 includes a representation of the extracellular space to account for the exchanges and transport systems connecting the extracellular space to the cytoplasm. The most recent community driven global reconstruction of human metabolism, Recon 2 (Thiele et al., 2013), is a substantial expansion over Recon 1 and includes more than 370 additional transport and exchange reactions/systems. These global reconstructions do not represent the metabolic capability of a single cell or tissue but rather are blueprints for all human cells. This reconstruction is analogous to the human genome, which encodes all of the cellular functions that may be active in one or more cell-types and conditions.

A reconstruction represents the metabolic repertoire of an organism or a cell in a condition-independent manner and can give rise to multiple condition-specific metabolic models. Consequently, recent COBRA modeling efforts have focused on generating cell-type specific metabolic models using cell-type and condition-specific data (e.g., transcriptomic and metabolomic data). Cell-type specific reconstructions have been assembled for cardiomyocytes (Zhao and Huang, 2011; Karlstaedt et al., 2012), hepatocytes (Gille et al., 2010; Jerby et al., 2010; Bordbar et al., 2011a), alveolar macrophages (Bordbar et al., 2010), red blood cells (Bordbar et al., 2011b), renal cells (Chang et al., 2010), enterocytes of the small intestine (Sahoo and Thiele, 2013), and different cancer cells (Agren et al., 2012; Jerby et al., 2012). In addition, the metabolic interactions among cell types, such as brain cells (Lewis et al., 2010) and hepatocytes, myocytes, and adipocytes (Bordbar et al., 2011a), have been modeled.

The generation of cell- and tissue-specific reconstructions requires extensive knowledge about the metabolites that can be transported across the plasma membranes of such cells. Transporters connect cells and tissues with their immediate environment and thus can be used to define the metabolite exchange pattern and, subsequently, which intracellular metabolic pathways involving these metabolites must be active to fulfill the chief functions of the cell or tissue. At the same time, transport reactions are amongst the least well studied reactions captured in Recon 1 (Duarte et al., 2007), and this is also the case with Recon 2, despite substantial efforts to include more transport information. This lack of information is mostly because the precise function and mechanism cannot be predicted from sequence data alone for transporter-encoding genes. We focus this review on plasma membrane transporters because they are generally better studied than intracellular transporters.

This review aimed to highlight the extent of our current knowledge about plasma membrane transporters and how well these data are captured in Recon 2. First, we introduce general transport mechanisms, and then we discuss the transport of five major metabolite classes (i.e., sugar, amino acids, lipids, vitamins, and others) based on their representation in Recon 2. Ions are also discussed, although they are not metabolites, because they are important co-substrates for many transport systems. All of the transport proteins are listed along with the unique NCBI EntrezGene identifiers (GeneID) of the encoding gene. At the end, we discuss the importance of transporters in different metabolic diseases and cancer. This review is accompanied by a transport module containing 70 new transport reactions that can be added to Recon 2. In addition, 24 transport reactions were identified within Recon 2 that need to be updated with the GPR associations only to capture the most current knowledge. These updates are summarized in Supplemental Table S2.

This review does not represent an update of Recon 2, but rather an expansion of its coverage of plasma membrane transport. The module-based approach permits researchers to actively contribute to the expansion of human metabolism, while maintaining Recon 2 as a core GENRE.

### General transport mechanisms

**Figure 1:**
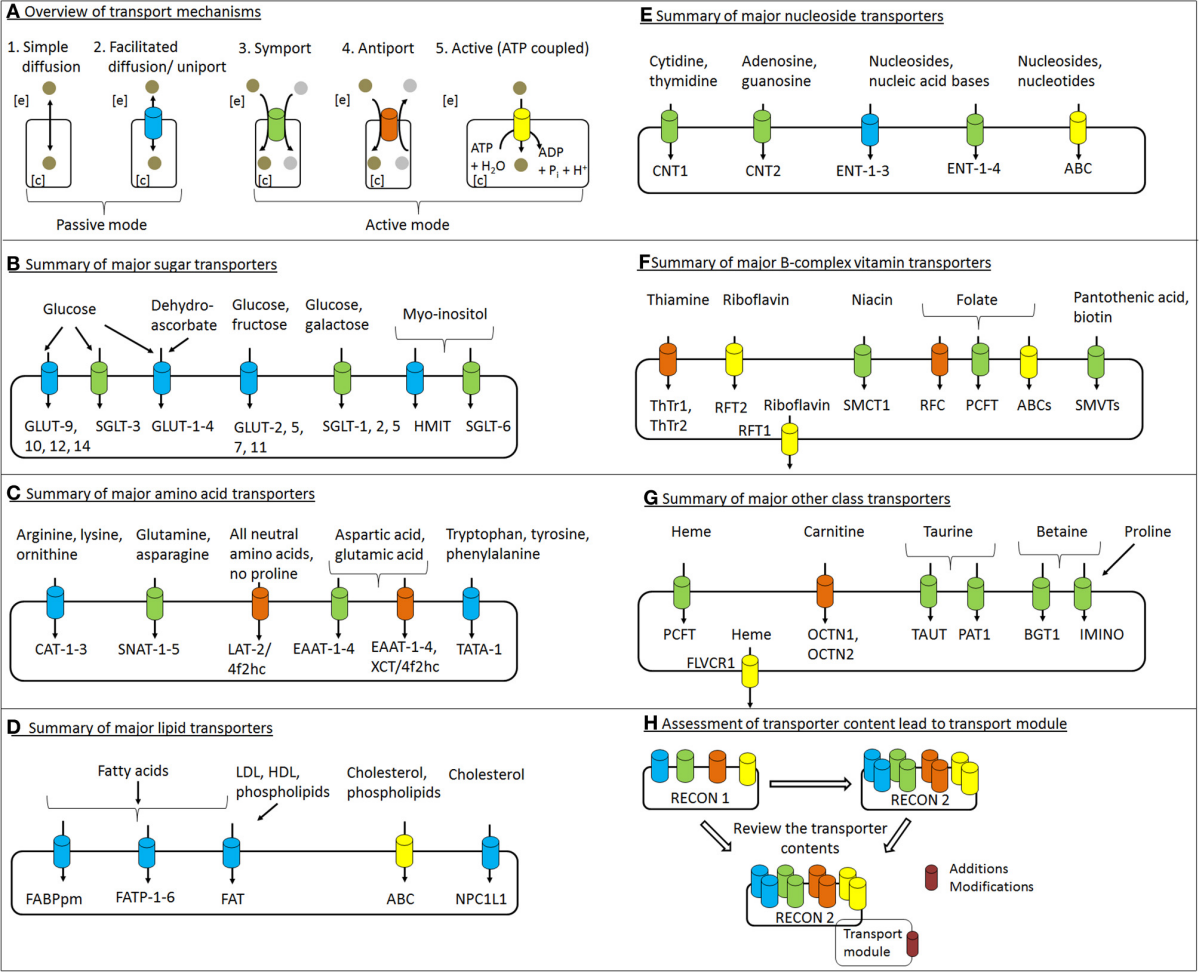
**Overview of transport mechanisms and major transport proteins of the various metabolite classes. (A)** The basic modes for metabolite transport across the plasma membrane are shown. Based on the energy association, transport processes can be categorized into active and passive modes based on the energy used. The active mode can be further classified into primary and secondary mechanisms, while the metabolites can also be transported mainly via simple diffusion or facilitated diffusion driven by an increase in entropy. Specialized transport mechanisms (e.g., receptor-mediated endocytosis and tertiary active processes) are not shown. **(B–G)**. Highlights major transport proteins involved in the transport of various substrates belonging to the sugar, amino acid, lipid, nucleoside, vitamin, and other classes mentioned in the text. **(H)** The present work accesses the coverage and gain in membrane transport systems with reference to the global human metabolic reconstruction, Recon 2 over Recon 1. The review of the relevant scientific literature led to the generation of transport reaction module that contained the proposed additions and modifications, discussed throughout the text. Refer to the text for a further explanation of these transport processes. The color coding for the transport mechanism as shown in **(A)** has been maintained in the other panels.

The cell membrane separates a cell from the extracellular environment. While hydrophobic substances can easily cross the lipid cell membrane by simple diffusion, hydrophilic substances cannot (Guyton and Hall, 2000). There are two basic modes of cellular transport for hydrophilic substances (i.e., active and passive). These basic mechanisms can operate as follows: (1) without a carrier protein (simple diffusion), (2) with a carrier protein (facilitated diffusion), and (3) with the expenditure of energy (primary and secondary active). The various modes of transport are shown in Figure 1A.

The cell membrane's lipophilicity defines the metabolites that can freely move in and out of the cell, a process called “simple diffusion.” Simple diffusion is directed from a region of high solute concentration to a region of low solute concentration. Various factors determine the net rate of diffusion, including the concentration difference of the solute, pressure difference between the cell and the environment, membrane electric potential, and osmosis (Guyton and Hall, 2000).

Transport processes allowing the passage of a single solute at a time are referred to as a uniport. “Facilitated diffusion” is an example of uniport transport (Lodish et al., 2000). In facilitated diffusion (also called carrier diffusion), the cargo molecule itself causes a conformational change in the carrier protein, which opens a channel for the cargo to cross the cell membrane. The capacity of this transport mechanism is thereby limited by the time needed to change the conformation back and forth. Facilitated diffusion occurs in both directions (Guyton and Hall, 2000).

Active transport is required to move molecules against their concentration and electrochemical gradients and requires energy in the form of ATP or other high energy phosphate bonds (Guyton and Hall, 2000). ATP hydrolysis is either directly connected to the transport (primary active transport) or is generated as an electrochemical gradient (secondary active transport) (Alberts et al., 2002; Forrest et al., 2011). When a secondary active transport process is further coupled to another distinct exchange mechanism, this process is referred to as tertiary active transport. One example is the coupling of amino acid transport system A (SNAT2) with system L (LAT1/4f2hc) for leucine uptake. SNAT2 utilizes the electrochemical gradient established by the Na^+^/K^+^ ATPase pump to drive its substrate into the cell (Baird et al., 2009).

Symport is the transport of multiple solutes across the cell membrane at the same time and in the same direction. If the inward transport of one solute is connected to the outward transport of another solute, the process is referred to as antiport (Alberts et al., 2002).

### Overview of extracellular transport reactions in human genres

**Figure 2:**
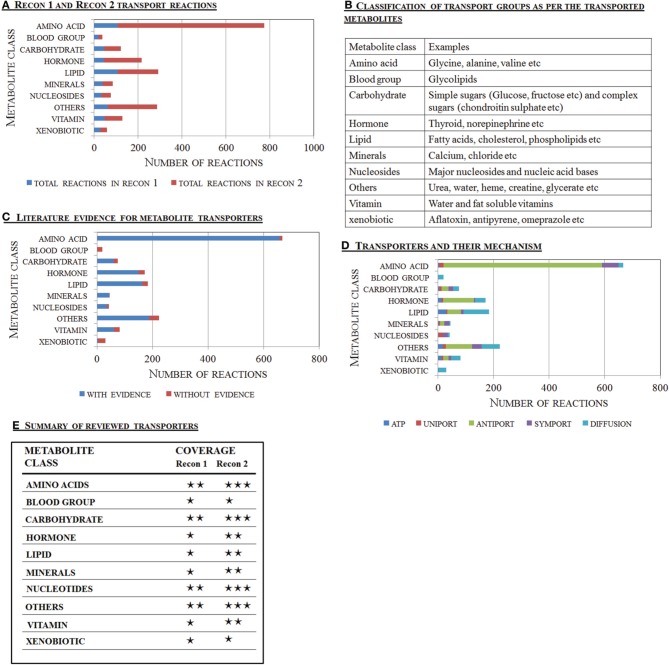
**Overview of transport reactions captured in the human metabolic reconstructions**. The transporter content for the major metabolite classes captured by Recon 2 and Recon 1 are shown and compared. **(A)** Quantitative assessment of the transport reactions present in Recon 1 (Duarte et al., 2007) and Recon 2 (Thiele et al., 2013). **(B)** Classification of the transport reactions as per the major class of metabolite transported. **(C)** Literature support for the transport reactions present in Recon 2. **(D)** The metabolites were divided into ten metabolite classes and their major transport mechanisms, as captured in Recon 2, are shown. The blood groups comprise the major glycolipids. **(E)** Comparison of the transport reactions present in Recon 1 and Recon 2. The increased information included and expanded the scope of Recon 2, over Recon 1, resulting in better transporter coverage for the amino acid, carbohydrate, and vitamin classes, while significant work is needed for the lipid class. The following symbols are used: ⋆⋆⋆, good coverage; ⋆⋆, intermediate coverage; ⋆, needs significant effort.

Recon 1 and Recon 2 are based on manually assembled biochemical knowledge, and their reactions are extensively annotated with literature evidence (Figure 2A). These GENREs contain 537 and 1537 extracellular transport reactions, respectively. The majority (89%) of the reactions in Recon 2 were supported by literature evidence to varying degrees. In this review, the metabolites are grouped into ten classes as in Recon 2 (Figure 2B). The amino acid class has the highest number of transport reactions, many of which have supporting evidence (Figure 2C). In contrast, the literature support for transport reactions in the “others,” “xenobiotics,” and “hormone” group was low. Such confidence gaps arise during the reconstruction process when information on the transport mechanism is not available, yet physiological evidence for the transport of a metabolite across the cell membrane has been reported or suggested (e.g., by body fluid or exo-metabolomic data). In such cases, the corresponding diffusion reactions are added to the reconstruction (Thiele and Palsson, 2010a). This lack of information regarding carrier proteins and mechanisms explains the high number of diffusion reactions for lipophilic metabolites falling into the “others,” “hormones,” and “lipids” groups in Recon 2 (Figure 2D). These gaps need to be filled as more knowledge is obtained (Figure 2E).

### Transport of sugars

Carbohydrates form a major part of the human diet. Polysaccharides, such as starch, are broken down into simple sugars in the intestinal lumen. Glucose, galactose, and fructose are the chief monosaccharides absorbed by enterocytes. From the enterocytes, sugars are released into the portal blood. Two main groups of sugar transporters exist, sodium/glucose co-transporters (SGLTs) and facilitated glucose transporters (GLUTs). Both groups are encoded by solute carrier (SLC) genes, where “SLC” is the initial official gene symbols (see Table 1). The SGLT (SGLT-1 to SGLT-6) family of transporters transport sugars coupled with sodium ions (secondary active transport). In contrast, the GLUT (GLUT-1 to GLUT-14) transporters mediate facilitated diffusion (Wu and Freeze, 2002; Wood and Trayhurn, 2003; Augustin, 2010). Some cells express both transporters for the uptake and secretion of simple sugars (see below).

**Table 1:**
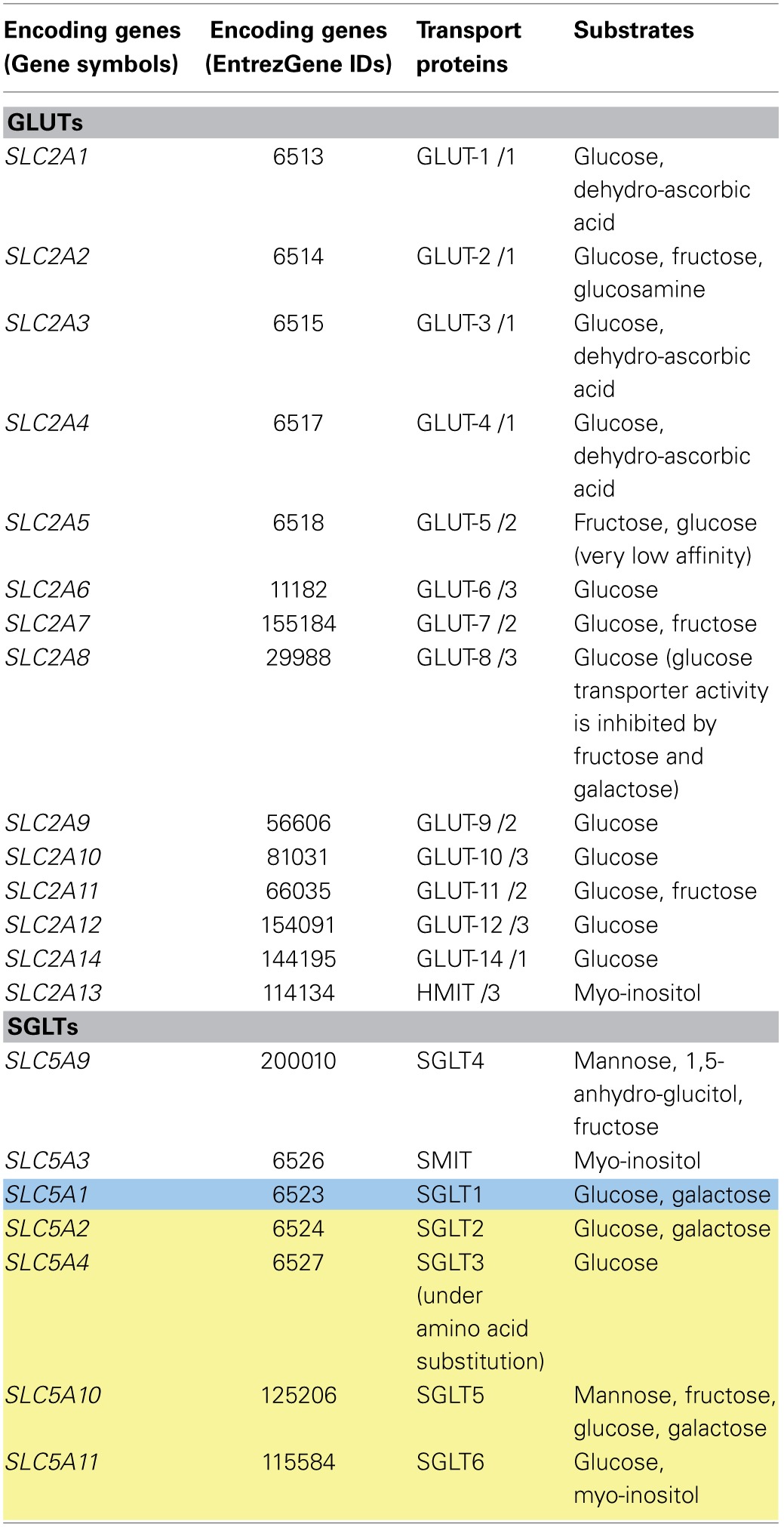
**Sugar transporters**.

*The data were assembled from Wood and Trayhurn (2003), Scheepers et al. (2004), & Thorens and Mueckler (2010). Yellow shading indicates genes encoding either absent transport proteins or transport proteins with limited substrate specificity in Recon 2. Blue shading indicates improvement in the transporter data (either the addition of the protein and its associated reactions, the expansion of its substrates, or modification of the GPRs) in Recon 2 over Recon 1. Transport proteins column also includes the corresponding GLUT class after the slash. Details of existent sugar transport reactions for Recon 2 are shown in Supplemental Table S1*.

#### SGLT transporters

SGLT-1 (*SLC5A1*, GeneID: 6523) is located on the apical side of enterocytes and renal tubules and mediates the influx of glucose and galactose via symport coupled with two sodium ions (Wood and Trayhurn, 2003; Gropper et al., 2009) (Figure 1B). The transporter is energized by the sodium gradient established by the Na/K-ATPase located on the basolateral surface (Hediger et al., 1995). However, under conditions of decreased luminal pH, proton-coupled glucose transport by SGLT-1 can take place (Thwaites and Anderson, 2007). The affinity of the transporter for glucose is reduced under these circumstances (Thwaites and Anderson, 2007). SGLT-2 (*SLC5A2*, GeneID: 6524)-mediated sugar re-absorption has been described in renal cells. SGLT-2 has a low affinity but high capacity for glucose and galactose transport (Hummel et al., 2011). SGLT-3, also called SAAT1 (*SLC5A4*, GeneID: 6527), is expressed in cholinergic neurons, small intestinal cells, and skeletal muscle cells. So far, the only confirmed function for SGLT-3 in humans is as a glucose sensor by depolarization of the membrane in the presence of high glucose (Diez-Sampedro et al., 2003). However, in *C. elegans*, a single amino acid substitution (from glutamate to glutamine) enables the protein to behave as a glucose transporter with transport properties similar to SGLT-1 (Bianchi and Diez-Sampedro, 2010). In addition, recent reports have claimed that SGLT-3 is a glucose-stimulated Na^+^ transporter (Kothinti et al., 2012). SGLT-4 (*SLC5A9*, GeneID: 200010) is a sodium-dependent mannose transporter, which also has affinity for 1,5-anhydro-glucitol and fructose (Tazawa et al., 2005). SGLT-4 is highly expressed in the small intestines and kidneys, but moderately in the liver (Tazawa et al., 2005). SGLT-5 (*SLC5A10*, GeneID: 125206) mediates the sodium-dependent uptake of sugars with the highest affinity for mannose, followed by fructose, and a very low affinity for glucose and galactose (Grempler et al., 2012). SGLT-5 is expressed in the kidneys. SGLT-6 (*SLC5A11*, GeneID: 115584) is a glucose and myo-inositol transporter. Compared to other tissues, SGLT-6 is the most highly expressed in the brain (Chen et al., 2010). The sodium myo-inositol co-transporter, SMIT (*SLC5A3*, GeneID: 6526), is expressed ubiquitously and at a high level in cells lining the blood vessels, the kidneys, and the thyroid gland (Chen et al., 2010). For a more elaborate tissue distribution for all of the SGLTs, see recent review by Wright et al. (2011).

#### GLUT transporters

The GLUT transporters have traditionally been divided into three families based on sequence similarity (Joost and Thorens, 2001; Scheepers et al., 2004) (Figure 1B). Wilson-O'Brien et al. proposed dividing the mammalian GLUT transporters into five distinct classes, subdividing class three proteins into three new classes [i.e., GLUT-6 and GLUT-8, GLUT-10 and GLUT-12, and the H^+^/myo-inositol transporter (HMIT)] (Wilson-O'Brien et al., 2010). Most of the GLUT transporters have been shown to transport glucose, with GLUT-2, GLUT-5, and GLUT-11 also transporting fructose, while HMIT also transports myo-inositol (Table 1). The transport of other non-carbohydrate substrates by the GLUT transporters has also been reported. Examples include uric acid transport by GLUT-9 (Doblado and Moley, 2009) and dehydroascorbate transport by GLUT-1 to GLUT-4 and GLUT-8 (Cura and Carruthers, 2012; Corpe et al., 2013). Only GLUT-4, GLUT-8, and GLUT-12 have been shown so far to exhibit insulin sensitivity (Wood and Trayhurn, 2003). Most of the GLUT transporters are expressed in the brain, which depends largely on glucose as an energy source (Vannucci et al., 1998; Bakirtzi et al., 2009).

#### Sugar transporters and Recon 2

The transport of sugars is generally well captured by Recon 2. In total, 80% (60/75 reactions) of the sugar transport reactions in Recon 2 are supported by literature evidence (Figure 2C). While the reactions for SGLT-1-mediated transport were added in Recon 2 (Gropper et al., 2009), the transport functions of the other SGLTs are still missing (Table 1). For instance, while SGLT-2-mediated glucose transport is captured in Recon 2, its galactose transport capability (Hummel et al., 2011) is not accounted for. Moreover, SGLT-5 is only associated with glucose transport, but not its additional substrates mannose, fructose, and galactose (Grempler et al., 2012). Finally, SGLT-6 is only associated with inositol but not glucose transport (Chen et al., 2010). The transport of galactose, mannose, fructose, and glucose already exists in Recon 2 with the correct transport mechanism; hence, only new genes have to be added to the GPRs of the corresponding reactions (see Table 1 and Supplemental Table S2).

### Transport of amino acids

Ingested proteins represent the body's main source of amino acids and peptides. Usually, multiple amino acids have the same transport protein (Table 2). The transport systems for both amino acids and peptides are discussed in this section.

**Table 2:**
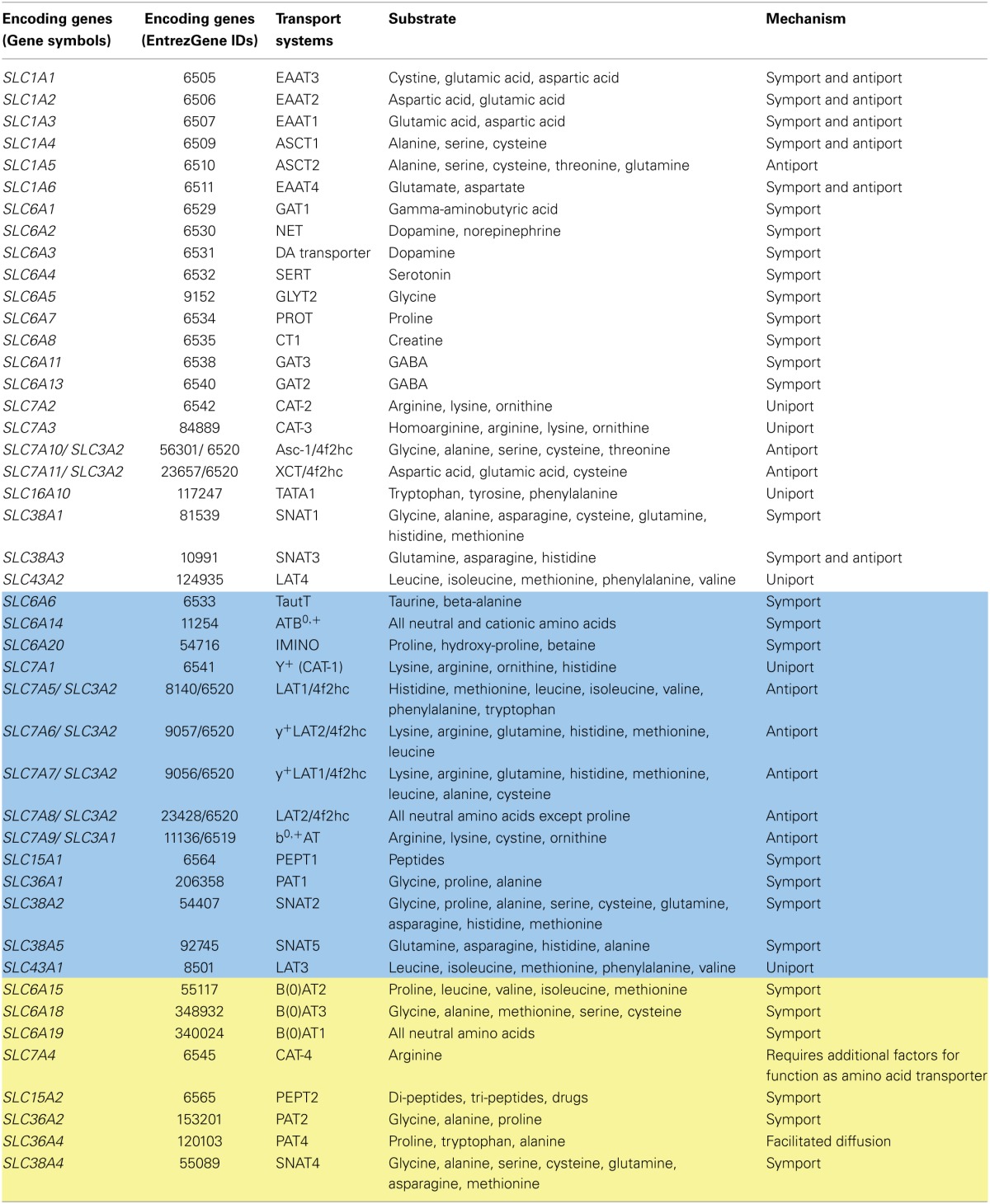
**Amino acid transport systems**.

*Important amino acid-derived compounds and peptides are also included. Color coding is the same as in Table 1. Details of the amino acid transport reactions in Recon 2 are shown in Supplemental Table S1*.

#### Amino acid transporters

Eleven different SLC families are known to be involved in the transport of amino acids either via antiport or symport (Broer and Palacin, 2011) (Figure 1C). There is considerable overlap in their substrate specificity (Table 2). Transporters in the SLC3 and SLC7 families form heteromeric protein complexes composed of heavy and light subunits (SLC3 genes encode the heavy subunit and SLC7 genes encode the light subunit of the transport protein) that interact through disulfide bridges. The heavy subunit is a glycosylated membrane protein; hence, these transporters are also called glyco-protein-associated amino acid transporters. The light subunit is required to confer specific amino acid transport activity (Palacin et al., 2005). The light subunit is fully functional even in the absence of the heavy subunit. The heteromeric amino acid transporters are usually amino acid exchangers (antiports) (Palacin et al., 2005). However, the alanine-serine-cysteine transporter (Asc-type amino acid transporter 1), a heteromeric amino acid transporter, with a heavy subunit encoded by *SLC3A2* (GeneID: 6520) and a light subunit by *SLC7A10* (GeneID: 56301), transports glycine, L- and D-alanine, L- and D-serine, threonine, and cysteine. This transport can be mediated either via facilitated diffusion or antiport. The antiport is the predominating mechanism (Fukasawa et al., 2000). The transporter is Na^+^/Cl^−^-independent and is found in the brain, heart, placenta, skeletal muscle, and kidneys (Nakauchi et al., 2000). In mice, this transporter was also identified in cells from the lungs and small intestines (Nakauchi et al., 2000).

Other members of the SLC7 family (SLC7A1-A4) are non-heteromeric proteins and cationic amino acid transporters (Figure 1C). The amino acids arginine, lysine, and ornithine are transported in a sodium-independent manner (Closs et al., 2006). While SLC7A1 is ubiquitously expressed, the SLC7A2 transporter (*SLC7A2*, GeneID: 6542) is found in skeletal muscle, placenta, ovary, liver, pancreas, kidneys, and heart (Hoshide et al., 1996). The SLC7A3 transporter (*SLC7A3*, GeneID: 84889) is expressed in brain, thymus, uterus, testis, and mammary glands (Vekony et al., 2001). The SLC7A4 transport protein (*SLC7A4*, GeneID: 6545) has no confirmed transporter activity; however, binding of the protein to other subunits to confer transport activity has been postulated (Wolf et al., 2002).

#### Peptide transporters

Four peptide transport proteins (Figure 1C) have been identified [i.e., PEPT-1 (*SLC15A1*, GeneID: 6564), PEPT-2 (*SLC15A2*, GeneID: 6565), hPHT1 (*SLC15A4*, GeneID: 121260), and hPHT2 (*SLC15A3*, GeneID: 51296)]. PEPT-1 and PEPT-2 are well-characterized proton symporters with overlapping substrate specificities. These symporters transport 400 distinct di-peptides, 8000 tri-peptides, and synthetically formulated drugs (Adibi, 2003). Generally, PEPT-2 exhibits a higher affinity for di- and tri-peptides than PEPT-1 (Shu et al., 2001; Biegel et al., 2006).

Peptide transporters are usually expressed on the apical side of polarized cells. While PEPT-1 is highly expressed in the small intestines, PEPT-2 is expressed in renal cells (Shu et al., 2001). All four peptide transport proteins have been identified in the nasal epithelium (Agu et al., 2011). Both PEPT-1 and PEPT-2 work via secondary active transport coupled to Na^+^/H^+^ exchange, where sodium ions are exported out of the cell via the basolateral Na^+^/K^+^ ATPase pump (Leibach and Ganapathy, 1996) to maintain the extra-cellular sodium concentration. The entire process is supported by intra-cellular peptide hydrolyzing enzymes. Peptides that escape hydrolysis are transported out of the cell via an uncharacterized basolateral peptide transporter (Pieri et al., 2010) that can mediate either facilitative transport (Terada et al., 1999) or proton mediated transport (Thwaites et al., 1993).

PEPT-1 is regulated by hormones and its substrates. In addition to peptides, PEPT-1 is activated by various amino acids, including lysine, arginine, and phenylalanine. Some hormones, such as insulin, can activate the basolateral peptide transporter, while others, such as leptin, epidermal growth factor, and thyroid hormone, inhibit the apical uptake of peptides by this transporter (Adibi, 2003). However, PEPT-2 activity was enhanced by minerals, such as copper, zinc, and manganese (Leibach and Ganapathy, 1996). Interestingly, PEPT-1, when expressed in enteroendocrine cells, is involved in hormone secretion and thus participates in nutrient sensing (Miguel-Aliaga, 2012). In addition, increased expression of this transport protein has been associated with various inflammatory conditions, such as ulcerative colitis and Crohn's disease. In contrast, PEPT-1 is not expressed in colonocytes under normal physiological conditions (Charrier and Merlin, 2006).

#### Amino acid and peptide transport systems and Recon 2

Amino acid and peptide transport systems are well described in the literature and in Recon 2. The 667 amino acid transporter reactions make up the largest group of extracellular transport reactions in Recon 2, and 98% of them are supported by literature evidence (Figure 2C). Recon 2 already covered the recent additions and modifications to the amino acid transport systems, which were identified during the reconstruction of the small intestinal epithelial cell (Sahoo and Thiele, 2013) and the liver (Gille et al., 2010).

Recon 2, however, still needs to be extended to account for current knowledge (Table 2). (i) Missing transported substrates need to be added, and our module provides the corresponding transport reactions. For example, the renal cell specific transport system SNAT4 (*SLC38A4*, GeneID: 55089) also transports cysteine and methionine (Broer, 2008; Broer and Palacin, 2011). Additionally, the transporters B(0)AT2, B(0)AT3, and PAT4 are missing along with the transport reactions for their substrates. (ii) GPRs for the existing transport reactions need to be expanded (Supplemental Table S2). The function of PAT2 can be added by expanding the GPRs of the appropriate reactions in Recon 2 (Supplemental Table S2). (iii) In the case of the PEPT-2 transporter, Recon 2 correctly captures its transport of the di-peptide Cys-Gly, but other substrates are missing, such as the tri-peptide Trp-Trp-Trp (Leibach and Ganapathy, 1996). However, the metabolic fate of these missing substrates is currently not captured in Recon 2; thus, the addition of the transport reactions would create gaps. Hence, we did not include these reactions in the transport module. For a detailed list of the endogenous and xenobiotic substrates for PEPT-2, refer to Biegel et al. (2006). Supplemental Table S1 is a comprehensive list of amino acid transporters and their properties.

### Transport systems for lipids

Lipids are essential for many biological processes. The major dietary lipids are triacylglycerol, phospholipids, and sterols. These dietary lipids are broken down into free fatty acids, mono-acylglycerols, and cholesterol, which are subsequently absorbed by cells (Gropper et al., 2009). Cholesterol and phospholipids are essential membrane constituents. Phospholipids and glycolipids form lung surfactants. Fat is stored within cells as triacylglycerols and break down into glycerol and fatty acids, which are a major source of energy for various cellular processes. Lipids also act as precursors for second messengers. Cholesterol acts as a precursor for steroid hormones and bile acids (Murray et al., 2009).

Due to their hydrophobic properties, the majority of lipids can freely diffuse across the cell membrane. Nevertheless, a number of alternative transport mechanisms exist (Figure 1D): (1) fatty acid transport proteins, including FATP1 (*SLC27A1*, GeneID: 376497), FATP2 (*SLC27A2*, GeneID: 11001), FATP3 (*SLC27A3*, GeneID: 11000), FATP4 (*SLC27A4*, GeneID: 10999), FATP5 (*SLC27A5*, GeneID: 10998), and FATP6 (*SLC27A6*, GeneID: 28965); (2) the membrane associated fatty acid transporters FABPpm (*GOT2*, GeneID: 2806) and fatty acid translocase FAT (*CD36*, GeneID: 948); (3) ATP binding cassette transporters; (4) various lipoproteins (i.e., chylomicrons, very low density lipoprotein, low density lipoprotein, and high density lipoprotein); and (5) intracellular lipid transporters, such as non-specific lipid-transfer protein (*SCP2*, GeneID: 6342), acyl CoA binding protein (*DBI*, GeneID: 1622), fatty acid binding proteins/ cytoplasmic fatty acid binding proteins [i.e., FABPc (FABP1-9)] (Gossett et al., 1996; Furuhashi and Hotamisligil, 2008), and various proteins that aid in membrane turnover via insertion of new lipids into pre-existing membranes (i.e., flippase, floppase, scramblase, and flip-flop) (Devaux et al., 2008; Sanyal and Menon, 2009). The presence of multiple additional transport mechanisms besides diffusion is explained by the essential role of lipids in the cell and the need to control their transport and distribution. In addition, the structural differences among fatty acids, mono-acylglycerol, and cholesterol necessitate distinct transport systems.

#### Fatty acid transport

Fatty acid transport proteins (FATPs) are a family of six transporters (Figure 1D) that mediate the influx of long chain fatty acids (>10 carbons in chain length), usually associated with a long chain fatty acid activating enzyme present on the membrane (acyl-CoA synthetases, E.C. 6.2.1.3) (Jia et al., 2007). FATPs have also been suggested to possess inherent fatty acid activating properties (Stahl, 2004), and they have an AMP-binding motif (Glatz et al., 2010). The membrane associated fatty acid transporters (FABPpm) also transport long chain fatty acids, although, compared to FATPs, FABPpms have a higher affinity toward long chain poly-unsaturated fatty acids and essential fatty acids (Dutta-Roy, 2000). The FABPpm transport mechanism is slightly different compared to FATP. Once transported inside the cell by FABPpm, fatty acids bind to the cytoplasmic counterpart (FABPc) and undergo activation (Glatz et al., 2010). The fatty acids might then be transported to other sub-cellular compartments by FABPc (Stewart, 2000). The fatty acid translocase protein (FAT, *CD36*, GeneID: 948) is usually expressed at low fatty acid concentrations. FAT binds and concentrates fatty acids at the cell surface and enhances their diffusion across the membrane. Alternatively, FAT can also deliver fatty acids to FABPpm (Glatz et al., 2010), and it has a wide substrate coverage, including low- and high-density lipoproteins and phospholipids (Stahl et al., 2001; Febbraio and Silverstein, 2007). Interestingly, the individual transport capacities (i.e., without any concertation) of FAT and FABPpm (including FATP) have been shown in the rat skeletal muscles for palmitate (C16:0), and these transport proteins also play a significant role in fatty acid oxidation (Nickerson et al., 2009). Therefore, Chabowski et al. (2007) proposed that the need for an association between FAT and FABPpm would arise during conditions of increased fatty acid oxidation to meet the increased substrate demands.

Various ABC transport proteins transport fatty acids, cholesterol, phospholipids, and cholesterol derivatives (bile acids) (Supplemental Table S1). ABC transporters generally conduct primary active transport, act as ion channels for chloride, or regulate the function of ATP-sensitive potassium channels (Glavinas et al., 2004). These transport proteins have a wide substrate spectrum, including drugs, lipid metabolites, hormones, heme, iron, peptides, nucleosides, and vitamins (see Supplemental Table S1 for details on substrate specificity, associated disorders, and references for all relevant ABC transporters). A number of the ABC transport proteins are functional monomers, while most of the other transport proteins require dimerization or binding to other proteins to gain complete functionality (e.g., ABCB2/TAP1, ABCB3/TAP2, four transporters from the ABCD sub-family, and five transporters from the ABCG sub-family) (Glavinas et al., 2004). The group of ATP binding cassette transporters comprises 48 transport proteins, categorized into six different families. Of the total number of transport proteins, 32 are located on the plasma membrane, and 13 are intracellular transport proteins (ABCA2, ABCB2, ABCB7-10, ABCC6, ABCD1-4, ABCG1, and ABCG4), while only three act at the plasma membrane and intracellular locations. These proteins are the ATP-binding cassette sub-family B member 6, (*ABCB6*, GeneID: 10058) located in the plasma membrane, Golgi apparatus, and lysosomes; the ATP-binding cassette sub-family A member 1, (*ABCA1*, GeneID: 19) located in the plasma membrane and the Golgi apparatus; and the ATP-binding cassette sub-family B member 5 (*ABCB5*, GeneID: 340273), whose location remains to be identified (Orso et al., 2000; Kiss et al., 2012).

#### Transport by lipoproteins and cholesterol transport

Lipoproteins are spherical components, containing a hydrophobic lipid core, amphiphilic lipids, and proteins with hydrophilic amino acid side chains on the surface (Nelson and Cox, 2000). Lipoproteins vary in their apolipoprotein (Apo) content, density, and lipid components. Chylomicrons are the largest lipoproteins, have the least density (i.e., <1.006 g/ml), and carry the highest fraction of triacylglycerols (Nelson and Cox, 2000). They are formed in the endoplasmic reticulum of small intestine cells and carry the lipid components of the diet into the lymph where they enter the blood via the left subclavian vein (Nelson and Cox, 2000). When passing through the blood capillaries, lipoprotein lipase (*LPL*, GeneID: 4023, E.C. 3.1.1.34) extracts the free fatty acids and releases them into muscle and adipose tissues. The liver takes up the remnant chylomicrons, where the excess fatty acids may be used to synthesize triacylglycerols, which are further transported into tissues as part of very low-density lipoprotein (VLDL). After removing the triglycerides, the unused VLDL or VLDL remnants, which are intermediate-density lipoproteins, are then either reabsorbed into the liver or form low-density lipoprotein (LDL). The small intestine and liver also form precursors for high-density lipoprotein (HDL) and release them into the circulation. HDL transport is also called reverse cholesterol transport. The major components transported by the four lipoprotein classes (Figure 1D) are (i) triacylglycerol by chylomicrons, (ii) phospholipids and triacylglycerol by VLDL, (iii, iv) cholesteryl esters and phospholipids by LDL and HDL (Nelson and Cox, 2000). In addition, all fat-soluble vitamins (vitamin A, D, E, and K) are also transported within chylomicrons, passing from the intestinal epithelial cells into the lymph (Reboul and Borel, 2011). The cellular uptake of cholesterol is also mediated by Niemann-Pick C1-like protein 1 (*NPC1L1*, GeneID: 29881) and SRB-I (*SCARB1*, GeneID: 949), where the latter takes up cholesterol from HDL (Ikonen, 2008; Reboul and Borel, 2011). In contrast, in the case of polarized cells, luminal efflux occurs through the ATP-binding cassette sub-family G member 5/ ATP-binding cassette sub-family G member 8 ABCG5/ABCG8 (*ABCG5*, *ABCG8*, GeneID: 64240 & 64241), and basolateral efflux is mediated by the ATP-binding cassette sub-family A member 1 ABCA1 (*ABCA1*, GeneID: 19) (Ikonen, 2008). The substrate specificities of these proteins have not been entirely resolved, and the exact transport mechanism needs further experimental support. According to the current understanding, NPC1L1 is a uniport and is recycled through an endocytic route (Reboul and Borel, 2011). Bi-directional transport has been indicated for SRB-I (Ikonen, 2008; Reboul and Borel, 2011). Still, for the majority of lipid transporters (including for fat-soluble vitamins), the precise transport mechanism with respect to directionality, coupled ions or other compounds, and substrate stoichiometry remain uncertain (Reboul and Borel, 2011).

#### Lipid transport systems and Recon 2

In Recon 2, the majority of reactions associated with lipid transport were simple diffusion reactions (91 of 183 reactions, Figure 2D), and 11% of the reactions were not supported by literature evidence (Figure 2C). The substrate coverage of the existing FATPs was increased in Recon 2 with the addition of long chain fatty acid transport reactions (Table 3). However, FAT (*CD36*, GeneID: 948) and FABPpm (*GOT2*, GeneID: 2806) are still missing in Recon 2 and are captured in the transport module.

**Table 3:**
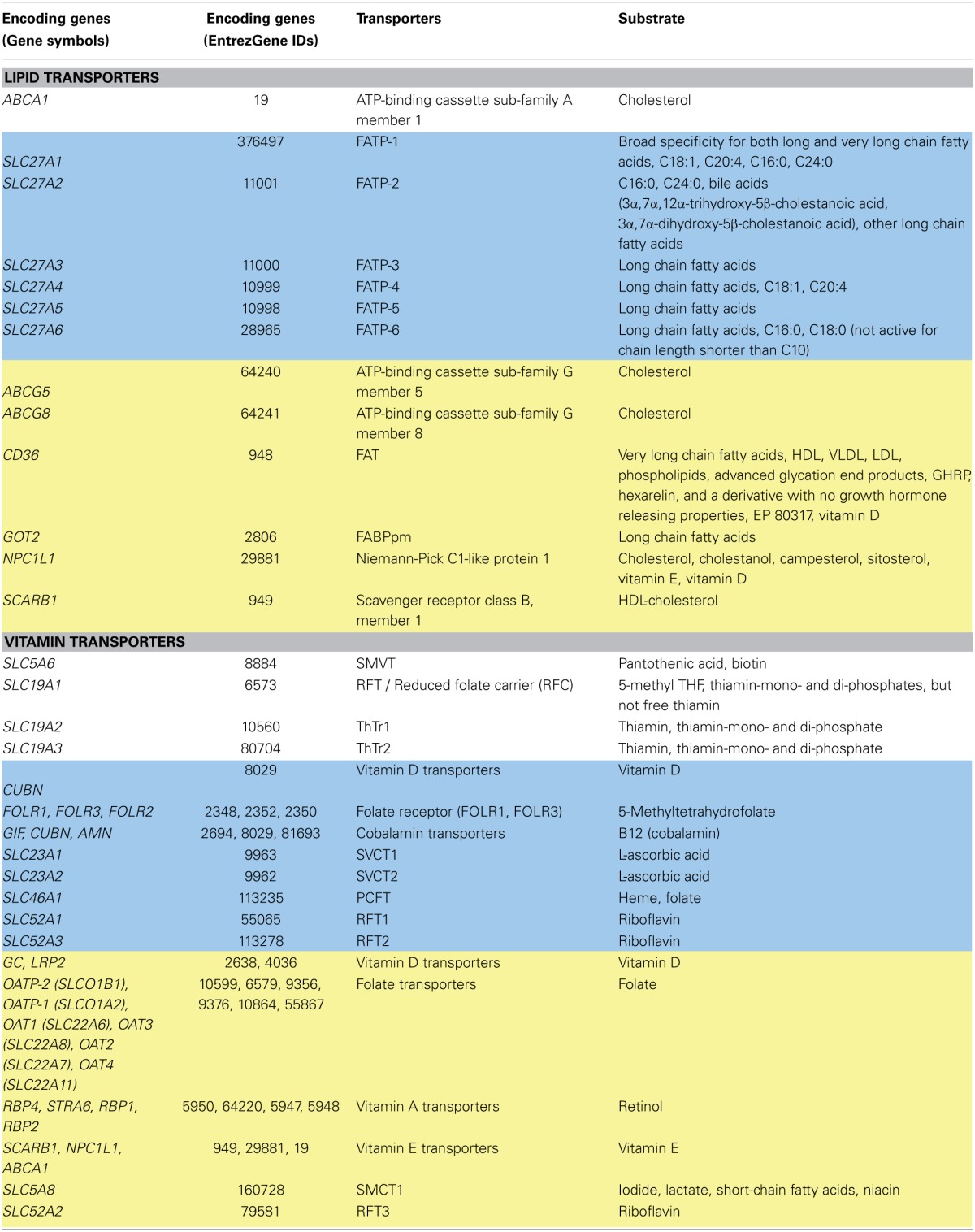
**Lipid and vitamin transporters**.

*The color coding is the same as in Table 1. Details of the lipid and vitamin transport reactions in Recon 2 are shown in Supplemental Table S1*.

Of all of the ABC transport proteins located at the plasma membrane, only seven transporters were captured in Recon 2. Moreover, the transport for a number of substrates is missing, which includes poly-unsaturated fatty acids, xenobiotics, nucleosides, nucleotides, and ions (see Supplemental Table S1 for a complete list). The transport module accounts for the missing transporters (ABCA3, ABCA4, ABCA8, ABCC11, and ABCG2). In addition, the transport module expands the substrate coverage for the ABCA1 transporter with phosphatidyl-choline and phosphatidyl-serine. Furthermore, the cholesterol transport proteins ABCG5 and ABCG8 are missing in Recon 2, and these proteins were added by expanding the GPRs for the corresponding reactions in Recon 2 (Supplemental Table S2).

### Transport system for nucleosides

The liver is the major organ for the de novo synthesis of all four nucleosides (Fustin et al., 2012). Another source of nucleic acids is ingestion and digestion. Nucleosides and nucleic acid bases are transported across biological membranes by concentrative nucleoside transporters (CNT) (Marce et al., 2006), equilibrative nucleoside transporters (ENT) (Marce et al., 2006), and transporters of the ABC transport family (Toyoda and Ishikawa, 2010; Fukuda and Schuetz, 2012).

Three CNTs exist (Figure 1E), each with distinct substrate specificity: CNT1 (*SLC28A1*, GeneID: 9154) shows a high affinity for pyrimidine nucleosides (e.g., cytidine, thymidine), CNT2 (*SLC28A2*, GeneID: 9153) prefers purine nucleosides (e.g., adenosine, guanosine), and CNT3 (*SLC28A3*, GeneID: 64078) exhibits a wide substrate specificity (Marce et al., 2006; Pastor-Anglada et al., 2007). CNTs mediate sodium-coupled secondary active symport. Recent findings have revealed the coupling of CNT3 with Na^+^ or H^+^ (Molina-Arcas et al., 2008). The four existing ENTs belong to the *SLC29A* gene family and exhibit a wide substrate specificity (Marce et al., 2006; Pastor-Anglada et al., 2007), including nucleic acid bases. ENT1-ENT3 mediate facilitated diffusion or uniport in a sodium-independent manner. ENT4 (*SLC29A4*, GeneID: 222962) works via a secondary active transport coupled to H^+^ (Molina-Arcas et al., 2008). In the case of enterocytes and renal cells, the CNTs are located at the apical surface, mediating the uptake of nucleosides, while ENTs mediate influx and efflux on the basolateral side (Pastor-Anglada et al., 2007). Both CNTs and ENTs transport a wide range of pharmacologically important drugs (Molina-Arcas et al., 2008). The transporters of the ABC transport family that transport nucleotides and nucleosides are multidrug resistance-associated protein 4 (*ABCC4*, GeneID: 10257), multidrug resistance-associated protein 5 (*ABCC5*, GeneID: 10057), ATP-binding cassette sub-family C member 11 (*ABCC11*, GeneID: 85320), and ATP-binding cassette sub-family G member 2 (*ABCG2*, GeneID: 9429) (Toyoda and Ishikawa, 2010; Fukuda and Schuetz, 2012). See Supplemental Table S1 for details on substrate specificity and associated properties.

#### Nucleoside transporters and Recon 2

Nucleoside transport is well established. Accordingly, 88% of the nucleoside transport reactions in Recon 2 are supported by literature evidence (Figure 2C). While only the reactions for the CNTs and ENT1-ENT3 are present in Recon 2, ENT4 (*SLC29A4*, GeneID: 222962) (Baldwin et al., 2004) was added to the transport module. Transport via CNT3 is associated with Na^+^ or H^+^ gradients (Molina-Arcas et al., 2008). However, only Na^+^-coupled secondary active transport was captured in Recon 2 (Supplemental Table S1). The CNT3-mediated, H^+^-coupled transport is covered in the transport module. In addition, gene information for the ABC transport proteins [i.e., ATP-binding cassette sub-family C member 11 (*ABCC11*, GeneID: 85320) and ATP-binding cassette sub-family G member 2 (*ABCG2*, GeneID: 9429)] needs to be added to the corresponding Recon 2 reactions (Supplemental Table S2). Another useful, future addition with respect to disease-directed research and the application of human GENREs could be the transport of nucleotide-derived drugs. Refer to (Molina-Arcas et al., 2008) for a list of drugs that are transported by these transporters.

### Transport system for vitamins

Vitamins are not synthesized by the human body and are therefore essential components of the human diet. Some vitamins, such as biotin, are also synthesized by the commensal gut microflora (Murray et al., 2009). Niacin can be synthesized in the body from the amino acid tryptophan (Murray et al., 2009). Vitamins have traditionally been divided into two groups: (1) fat-soluble vitamins comprising vitamins A, D, E, and K, and (2) water-soluble vitamins comprising the B complex of thiamin (B1), riboflavin (B2), niacin (B3), pyridoxal (B6), folic acid, cobalamin (B12), pantothenic acid, and biotin, and vitamin C. Vitamins play a major role in a variety of biochemical processes. Vitamin A is involved in the visual cycle, vitamin D in calcium metabolism, vitamin E in free radical scavenging, vitamin K in blood clotting, B1 in carbohydrate metabolism and nerve conduction, B2 and B3 in redox reactions, B6 in transamination reactions, folic acid and cobalamin in one carbon metabolism, pantothenic acid in fatty acid metabolism, biotin in carboxylation reactions and vitamin C in hydroxylation reactions (Murray et al., 2009). The body's inability to synthesize vitamins and their pivotal role in metabolic processes necessitate transport mechanisms other than simple diffusion for their import into a particular cell type and efflux for utilization by other cell types. Epithelial cells in the small intestine, kidneys, and liver express the vast majority of ABC and SLC transporters because these organs play a chief role in the absorption and secretion of endogenous metabolites and xenobiotics (Brunton et al., 2006; Klaassen and Aleksunes, 2010). Epithelial cells are among the best models to study transport processes. Hence, we will focus on the transport proteins present in the enterocytes of the small intestine.

### Transport of fat-soluble vitamins (FSVs)

FSVs were believed to enter enterocytes from the intestinal lumen via passive diffusion. However, transport proteins for vitamins A, D, and E have been identified, and energy-dependent transport has been suggested for vitamin K in rats (Hollander et al., 1977).

Vitamin A is transported in the plasma bound to retinol binding protein. Its uptake into enterocytes is mediated by retinoic acid gene 6 protein homolog (STRA6) protein (*STRA6*, GeneID: 64220) (Berry et al., 2011). A transporter of ABC family [i.e., retinal-specific ATP-binding cassette transporter (*ABCA4*, GeneID: 24)], is involved in the transport of retinaldehyde, a form of vitamin A, into retinal photoreceptors (Burke and Tsang, 2011). Retinol-binding protein 1 (*RBP1*, GeneID: 5947) and retinol-binding protein 2 (*RBP2*, GeneID: 5948) aid in apical uptake, esterification, and secretion of retinol (Harrison, 2005). So far, no basolateral transport protein for vitamin A has been identified (Reboul and Borel, 2011). The basolateral efflux of retinol (in the form of retinyl-esters) has been assumed to primarily occur via chylomicrons.

Vitamin D is mainly transported in the circulation bound to vitamin D-binding protein, which has a higher affinity for 25-hydroxy vitamin D than for vitamin D3 (Ball, 2006). Once bound, the complex is recruited by megalin (*LRP2*, GeneID: 4036) and cubilin (*CUBN*, GeneID: 8029) either for hydroxylation or efflux (Dusso et al., 2005). In addition, SR-BI (*SCARB1*, GeneID: 949), FAT (*CD36*, GeneID: 948) and NPC1L1 (*NPC1L1*, GeneID: 29881) are involved in the intestinal uptake of vitamin D (Reboul et al., 2011). The SR-BI protein has been shown to play a role in the apical uptake and basolateral efflux of vitamins D and E in caco-2 cells (Reboul and Borel, 2011). Moreover, vitamin D is transported within chylomicrons into the lymph (Ball, 2006).

Vitamin E uptake appears to be similar to that for cholesterol. Although passive diffusion has been observed, additional receptor-mediated transport is through SRB-I and NPC1L1 (Reboul et al., 2006; Narushima et al., 2008). Basolateral efflux occurs via the ABC family protein ABCA1 (*ABCA1*, GeneID: 19) (Rigotti, 2007).

### Transport of water-soluble vitamins

Both the low concentration and hydrophilicity of water-soluble vitamins make simple diffusion highly inefficient. Distinctive carrier-dependent transporters exist at the apical and basolateral sides of enterocytes to mediate vitamin exchange (Figure 1F).

#### Vitamin B1

Three transport proteins are associated with the transport of vitamin B1, and its structural analogs [i.e., ThTr1 (*SLC19A2*, GeneID: 10560), ThTr2 (*SLC19A3*, GeneID: 80704), and RFT (*SLC19A1*, GeneID: 6573)]. While ThTr1 and ThTr2 can transport free thiamin, RFT transports the mono- and di-phosphate forms of thiamin (Zempleni et al., 2007; Said, 2011). Sub-cellular locations vary between the transporters. ThTr1 is located at the apical and basolateral membranes. In contrast, ThTr2 and RFT are localized only to the basolateral membrane. The transport of vitamin B1 occurs against concentration and an outwardly directed H^+^ gradient and appears to be sodium-independent, electroneutral, and pH-dependent (Said, 2004). However, the directionality or reversibility of these transport processes remains to be elucidated.

#### Vitamin B2

The uptake and secretion of vitamin B2 from enterocytes involves primary active transport (Said et al., 1993; Bates, 1997; Subramanian et al., 2011) mediated by three transport proteins, RFT1 (*SLC52A1*, GeneID: 55065), RFT2 (*SLC52A3*, GeneID: 113278), and RFT3 (*SLC52A2*, GeneID: 79581). RFT1 is expressed at the basolateral membrane and RFT2 at the apical membrane, leading to vitamin B2 efflux and uptake, respectively (Subramanian et al., 2011). Both transporters are expressed in the small intestine (Yao et al., 2010). RFT3 is specifically expressed in the brain (Yao et al., 2010).

#### Vitamin B3

The cellular uptake of niacin (also called vitamin B3 or nicotinic acid) can occur by simple diffusion (Ball, 2006). In addition, niacin is taken up via sodium-independent and temperature- and acidic pH-dependent facilitated diffusion (Nabokina et al., 2005). No specific carrier protein has so far been identified for vitamin B3. Yet, SMCT1 (*SLC5A8*, GeneID: 160728), principally an iodide transporter, has been suggested to mediate sodium-coupled niacin transport (Gopal et al., 2007). The mechanism for the basolateral efflux of niacin and the carrier protein involved is unknown (Said, 2011).

#### Vitamin B6

Vitamin B6 diffuses freely across the cell membrane (Ball, 2006). However, carrier-dependent transport (sodium-independent but pH-, energy-, and temperature-dependent) has also been suggested (Said et al., 2003). No specific transport protein has been characterized at the molecular level.

#### Folate (vitamin B9)

Folate plays a role in various biochemical processes (e.g., DNA synthesis, one carbon metabolism, and amino acid metabolism), in the prevention of congenital abnormalities (e.g., neural tube defects, urogenital abnormalities, cardiovascular malformations, cleft lip, and palate) and the prevention and treatment of cardiovascular diseases (Tolarova, 1982; Czeizel and Dudas, 1992; Czeizel, 1996; Tian and Ingwall, 2008). Given its general importance, not surprisingly, multiple folate transporters exist. Traditionally, the two folate carriers are the reduced folate carrier (*SLC19A1*, GeneID: 6573) and proton-coupled folate transporter (*SLC46A1*, GeneID: 113235). In addition, three high-affinity folate receptors, a number of ABC transporters, and members of the solute carrier organic anion transporter family have been associated with the transport of folate or its derivatives. They will be discussed in the following paragraphs.

The reduced folate carrier is an organic anion antiporter that utilizes a high trans-membrane organic phosphate gradient. This carrier is expressed at the distal part of the small intestine and operates at neutral pH (Said, 2011). The proton-coupled folate transporter mediates the transport of folic acid and 5-methyl- and formyl-tetrahydrofolates. The transporter localizes to the proximal small intestine, operates at an acidic pH (Said, 2011; Zhao et al., 2011), and has been shown to also transport heme (Zhao et al., 2011).

The three high-affinity folate receptors are FRα (*FOLR1*, GeneID: 2348), FRβ (*FOLR2*, GeneID: 2350), and FRγ (*FOLR3*, GeneID: 2352). They mediate the unidirectional influx of folate, whereby the entire folate-receptor complex is internalized (Ball, 2006; Zhao et al., 2011). A reversible transport has also been suggested (Zempleni et al., 2007).

The basolateral folate transporter has not yet been characterized at the molecular level. However, the presence of a specific carrier protein mediating sodium-independent but pH-dependent folic acid transport has been shown in rats (Hamid et al., 2009). In the case of humans, 5-methyl-tetrahydrofolate has been identified in the portal blood (Ball, 2006), but the corresponding transport mechanisms and proteins have not been elucidated.

Seven ABC transporters expressed in the plasma membrane in different epithelial and non-epithelial cells have shown affinity toward folate and its derivatives (Matherly and Goldman, 2003; Toyoda and Ishikawa, 2010; Zhao et al., 2011). Multidrug resistance-associated protein 1 (*ABCC1*, GeneID: 4363) and multidrug resistance-associated protein 5 (*ABCC5*, GeneID: 10057) are expressed on the basolateral side, and the canalicular multispecific organic anion transporter 1 (*ABCC2*, GeneID: 1244) is present on apical side of enterocytes. *ABCC5* and *ABCC2* are further expressed on the basolateral side of hepatocytes. The remaining four ABC transporters are ATP-binding cassette sub-family G member 2 (ABCG2, GeneID: 9429), multidrug resistance protein 3 (*ABCB4*, GeneID: 5244), ATP-binding cassette sub-family C member 11 (*ABCC11*, GeneID: 85320), and multidrug resistance-associated protein 4 (*ABCC4*, GeneID: 10257).

Numerous solute carrier organic anion transporters (OAT) transport methotrexate (a structural analog of folic acid) and are also relevant folate transporters in the liver and kidneys (Matherly and Goldman, 2003; Zhao and Goldman, 2003). These transporters include OATP-2 (*SLCO1B1*, GeneID: 10599) in the liver (basolateral side) and OATP-1 (*SLCO1A2*, GeneID: 6579), OAT1 (*SLC22A6*, GeneID: 9356), OAT3 (*SLC22A8*, GeneID: 9376), OAT2 (*SLC22A7*, GeneID: 10864), and OAT4 (*SLC22A11*, GeneID: 55867) in the kidneys (Takeda et al., 2002; Badagnani et al., 2006; Zhao et al., 2011). One can refer to (Zhao et al., 2011) for the precise apical/basolateral localizations of these folate transporters.

#### Vitamin B12

Vitamin B12 is the precursor for two coenzymes, adenosylcobalamin and methylcobalamin. Adenosylcobalamin is required for methylmalonyl CoA-mutase activity (E.C. 5.4.99.2), which catalyzes the conversion of methyl malonyl-CoA to succinyl-CoA. Methylcobalamin is required for methionine synthase activity (E.C. 2.1.1.13), which catalyzes the methylation of homocysteine to methionine (Murray et al., 2009; Watkins and Rosenblatt, 2011). The absorption of cobalamin by simple diffusion along the entire small intestine accounts for 1–3% of dietary vitamin B12. Interestingly, this vitamin depends on a carrier-mediated transport when administered in pharmacological doses (Ball, 2006).

Cobalamin is transported into intestinal epithelial cells by cubilin-mediated absorption. The vitamin binds to intrinsic factor (*GIF*, GeneID: 2694), building the intrinsic factor-cobalamin complex (IF-Cbl) and to two proteins called cubilin (*CUBN*, GeneID: 8029) and amnionless (*AMN*, GeneID: 81693). The latter serve as an anchor for the receptor and aids cobalamin uptake. In addition, proteins, such as megalin and receptor-associated protein, can interact with CUBN. Whether the binding of additional proteins plays a role in the CUBN-mediated absorption of IF-Cbl has not been determined (Quadros, 2010). The protein responsible for the basolateral efflux of cobalamin has not been experimentally validated. However, multidrug resistance-associated protein 1 (*ABCC1*, GeneID: 4363) has been shown to transport cobalamin in prokaryotes and eukaryotes, including mice (Green, 2010).

#### Pantothenic acid and biotin

Pantothenic acid and biotin are absorbed at the apical membrane by a common sodium coupled symporter, SMVT (*SLC5A6*, GeneID: 8884) (Ball, 2006). In addition, SMVT transports lipoic acid and is called a multi-vitamin transporter. The basolateral release of biotin is mediated by a yet uncharacterized carrier protein in a sodium-independent manner (Said, 1999). The mechanism of basolateral pantothenate efflux remains to be elucidated (Said, 2011).

#### Vitamin C

Two transport proteins, the apically located SVCT1 (*SLC23A1*, GeneID: 9963) and the basolaterally located SVCT2 (*SLC23A2*, GeneID: 9962), mediate vitamin C (also called ascorbate or L-ascorbic acid) uptake. The membrane location has been confirmed in rats (Boyer et al., 2005). SVCT2 is ubiquitously expressed (except in the lungs and skeletal muscle), whereas SVCT1 is confined to the intestines, liver, kidneys, colon, ovaries, and prostrate (Liang et al., 2001). Both transport proteins exhibit Na^+^-coupled secondary active symport (coupling ratio 2:1) (Liang et al., 2001), energized by Na^+^/K^+^ ATPase (Ball, 2006). Ascorbate export has been assumed via volume-sensitive anion channels (Wilson, 2005). Alternatively, intracellular vitamin C can be oxidized to dehydroascorbate, which can freely diffuse into the blood stream (Gropper et al., 2009). Three transporters in the GLUT family (GLUT-1, GLUT-3, and GLUT-4) also mediate the uptake of dehydroascorbate on the basolateral side (Wilson, 2005). In astrocytes, GLUT-1 facilitates entry, and GLUT-3 mediates the efflux of dehydroascorbate (Hediger, 2002).

#### Vitamin transporters and Recon 2

Transport systems for water-soluble vitamins have been more intensively investigated than FSVs (Reboul and Borel, 2011). FSV transport was not well represented in Recon 2, but the transport of water-soluble vitamins was fairly well captured. Overall, 74% of the vitamin transport reactions are supported by literature evidence (Figure 2C). However, the genes encoding for proteins transporting fat-soluble vitamins, including those discussed for vitamins A, D, and E (i.e., STRA6, ABCA4, RBP1, RBP2, LRP2, CUBN, SR-BI, and NPC1L1), are absent in Recon 2. The transport protein encoded by the *ABCA1* gene is so far only associated with cholesterol, but not vitamin E transport (Table 3). The transport module accounts for vitamin A transport by ABCA4, while the other missing genes have been added by expanding the GPRs of the respective transport reactions (Supplemental Table S2).

Recon 2 includes the vitamin B2 transporters, RFT1 and 2, but not RTF3, which can be added by expanding the corresponding GPRs (Supplemental Table S2). Recon 2 also accounts for the substrate specificity of PCFT, FOLR1, and FOLR3. The transport of folate by FOLR2, and of vitamin B3 by SMCT1 can be accounted for by expanding the GPR of the corresponding reaction. OAT1—OAT4-mediated transport can be added, via the module, to completely capture the current knowledge about folate transporters. The vitamin B12 transport proteins (i.e., intrinsic factor, cubilin, and amnionless) and the ATP costs of SVCT1/SVCT2 transport are already accounted for in Recon 2. See Supplemental Table S1 for the vitamin transporters and their properties.

### Transport of water, heme, and other special compounds

Water moves across biological membranes via different mechanisms. Apart from diffusing through the lipid bilayer, co-transporters in the form of protein channels exist in the membrane, through which water can diffuse. The movement of water molecules through such channels, called aquaporins, is driven by osmosis (Macaulay et al., 2004). Water is also a substrate for co-transporters, such as excitatory amino acid transporter 1 EAAT1 (*SLC1A3*, GeneID: 6507), which is expressed in the brain and moves both urea and water along with glutamate (Vandenberg et al., 2011), and for the sodium glucose co-transporter, SGLT1, which transports sodium and glucose, while causing water influx (Zeuthen et al., 2001). For details on the various water co-transporters, specifically those operating in the brain, one may refer to (Macaulay et al., 2004).

#### Aquaporins

Aquaporins are a family of membrane channel proteins that allow the passage of water molecules, neutral molecules (e.g., urea and glycerol) and other small solutes (Zardoya and Villalba, 2001). In total, 13 members of this family have been characterized at the molecular level, and they are expressed in a wide variety of tissues (abundantly in the epithelial layer of the kidneys, intestine, lungs, and brain) (Verkman, 2005). While a majority of these proteins are expressed on the plasma membrane, aquaporin-6 (*AQP6*, GeneID: 363) and aquaporin-2 (*AQP2*, GeneID: 359) are also localized to intracellular vesicles (Yasui et al., 1999; Verkman, 2012). Interestingly, these proteins have been associated with various cellular functions, including skin hydration (Dumas et al., 2007), neural signal transduction (Amiry-Moghaddam et al., 2003; Yang et al., 2013), and cell volume regulation (Hansen and Galtung, 2007). Aquaporins are believed to hold therapeutic potential for congestive heart failure, hypertension, glaucoma, brain swelling, epilepsy, obesity, and cancer (Verkman, 2005, 2012; Tradtrantip et al., 2009).

#### Heme

Heme forms the prosthetic group of hemoglobin and other heme-containing proteins, such as myoglobin, cytochromes P450, cytochrome C, tryptophan pyrrolase, and catalase (Murray et al., 2009). In addition, heme degradation serves as a source for the essential micronutrient iron (Iannotti et al., 2006). Two transport proteins have been identified for heme (Figure 1G), the proton-coupled folate transporter (*SLC46A1*, GeneID: 113235, discussed above) and the feline leukemia virus subgroup C receptor-related protein 1 (*FLVCR1*, GeneID: 28982). These transport proteins directly transfer extracellular heme into the cell. While the proton-coupled folate transporter acts at the apical surface, the feline leukemia virus subgroup C receptor-related protein 1 is believed to have an active transport mechanism (Uc et al., 2004) and is localized to the basolateral surface of polarized cells (West and Oates, 2008). The hemopexin protein directly interacts with feline leukemia virus subgroup C receptor-related protein 1, hence increasing heme efflux, which is perceived to be a cellular protection against heme toxicity (Yang et al., 2010). Heme transport can also occur via receptor-mediated endocytosis, by prolow-density lipoprotein receptor-related protein 1 (*LRP1*, GeneID: 4035), which has been proposed to play a role in inflammation (Hvidberg et al., 2005). The ABC transporter ATP-binding cassette sub-family G member 2 (*ABCG2*, GeneID: 9429) can also transport heme (Krishnamurthy et al., 2004).

### Transport of conditionally essential nutrients

In addition to essential nutrients, there are certain other conditionally essential nutrients (CEN), which are usually synthesized by the body in almost sufficient amounts. However, under conditions of increased need, such as tissue injury or neonatal conditions, these nutrients may need to be derived from the diet. CEN includes compounds, such as arginine, CoQ10, carnitine, propionyl carnitine, taurine, lipoic acid, betaine, ribose, cysteine, chondroitin sulfate, and glutamine (Kendler, 2006; Soghier and Brion, 2006). In this section, we will focus only on carnitine, taurine and betaine because the transport of arginine, cysteine, glutamine, and ribose has already been discussed in the relevant sections above (also see Supplemental Table S1).

#### Carnitine

Carnitine transports fatty acyl-CoAs (i.e., activated fatty acids) into mitochondria, via the carnitine shuttle system (Murray et al., 2009) (Figure 1G). A positive effect of carnitine supplementation has been demonstrated for neuro-regeneration in rats (McKay Hart et al., 2002), liver cirrhosis in children (Selimoglu et al., 2001), obesity and associated metabolic disorders (Amin and Nagy, 2009), congestive heart failure (Kobayashi et al., 1992), and various other diseases, which are reviewed in Flanagan et al. (2010). Carnitine further exerts protective effects in corneal epithelial cells preventing the deleterious effects of dry eye syndrome (Xu et al., 2010). While carnitine synthesis occurs using methionine and lysine in the liver and kidney (Flanagan et al., 2010), exogenous carnitine needs transporters to reach the target cells. There are two membrane transport proteins for this purpose, OCTN1 (*SLC22A4*, GeneID: 6583) and OCTN2 (*SLC22A5*, GeneID: 6584). OCTN1 transports organic cations and carnitine (in zwitter ion form) in a pH-dependent and sodium-independent manner, chiefly behaving as a proton/organic cation antiporter at the apical surface of polarized cells (Yabuuchi et al., 1999). OCTN2 is a sodium-dependent carnitine transporter that is also localized on the apical membrane and mediates organic cation/carnitine exchange (Ohashi et al., 2001). Additional carnitine transport proteins are the amino acid transporter ATB^0,+^ (*SLC6A14*, GeneID: 11254), which we discussed above (Hatanaka et al., 2004), and CT2 (*SLC22A16*, GeneID: 85413), which is exclusively found in the testis and functions in a sodium-independent manner (Enomoto et al., 2002). ATB^0,+^ operates when OCNT2 is defective (Srinivas et al., 2007).

#### Taurine

One of the end products of methionine and cysteine metabolism is taurine, which plays an important role in a number of tissues. In the brain, taurine acts as a neuromodulator, neurotransmitter, and membrane stabilizer (Tamai et al., 1995). High taurine concentrations in the heart and muscles support its contractile function and osmo-regulation, and taurine can also exert antioxidant action by neutralizing hypochlorous acid and regulating mitochondrial protein synthesis in these tissues (Schaffer et al., 2010). Additional evidence for the importance of this amino acid in human health suggests its positive effect on growth in low birth weight infants, promotion of biliary flow, and prevention of cholestasis (Guertin et al., 1991; Stapleton et al., 1997). Disruption of taurine transport causes retinal degeneration in mice (Heller-Stilb et al., 2002). Two taurine transporter exist (Figure 1G), TAUT (*SLC6A6*, GeneID: 6533) and PAT1 (*SLC36A1*, GeneID: 206358). TAUT (*SLC6A6*, GeneID: 6533) mediates sodium and chloride ion-coupled secondary active transport. The stoichiometry is 1 taurine: 2 sodium: 1 chloride, but limited transport activity has also been observed without chloride (Tamai et al., 1995). Although the transport directionality remains to be confirmed, the movement of taurine through the blood-brain barrier was shown to occur from the blood into the brain (Tamai et al., 1995). The second taurine transporter PAT1 (*SLC36A1*, GeneID: 206358) operates via H^+^/taurine symport. This high-capacity but low-affinity transporter, which also transports beta-alanine, is highly expressed on the apical membrane of enterocytes (Anderson et al., 2009).

#### Betaine

Betaine is another important molecule involved in methionine metabolism. Once synthesized from choline, betaine donates its methyl group to regenerate methionine from homocysteine and helps to conserve the cellular methionine level (Craig, 2004). In addition, betaine acts as an osmolyte, particularly helpful for normal physiological functions of the kidneys, intestinal epithelium, red blood cells, and skeletal muscle. Moreover, its protective role has been observed in the heart and liver cells (Craig, 2004). Na^+^/Cl^−^-dependent secondary active betaine transport (Figure 1G) is mediated by BGT-1 (*SLC6A12*, GeneID: 6539) (Yamauchi et al., 1992). Another study reported Na^+^-independent, passive transport in rats (Craig, 2004). An alternate substrate of BGT-1 is gamma-amino-butyric acid (see Supplemental Table S1). Details regarding the directionality of BGT-1-mediated transport remain unknown. The amino acid transporter imino (*SLC6A20*, GeneID: 54716) also transports betaine (Broer, 2008).

#### Transport of water, heme and other special compounds in Recon 2

Recon 2 contain aquaporin-8 (*AQP8*, GeneID: 343) and aquaporin-9 (*AQP9*, GeneID: 366) for the transport of water, urea, and lactate. Extracellular water transport also occurs in Recon 2 through simple diffusion (“H2Ot”) and co-transport via SGLT-1 (“UREAt5”). The other aquaporins (AQP0, AQP1- AQP5, AQP7, and AQP10) need to be added (see Supplemental Table S1 for details on water transporters and their associated properties). The transport module adds reactions and genes for AQP3, AQP7, and AQP10. The remainder of the aquaporins can be accounted for by expanding the GPRs of the corresponding reactions (Supplemental Table S2). Recon 2 lacks the heme transporter FLVCR1 because additional biochemical experiments needed to clarify the precise transport mechanism. LRP1 can be added by GPR modification (Supplemental Table S2). All of the above discussed carnitine transport proteins, except for CT2, are present in Recon 2. The function of CT2 is captured in the transport module. In addition, carnitine transport mediated by the amino acid transporter ATB^0,+^ is missing in Recon 2 but can be accounted for through the transport module. Efficient taurine transport, via TAUT, coupled to Na^+^ and Cl^−^ ions, is present in Recon 2. The transport reactions catalyzed by BGT-1 [i.e., “ABUTt4(2)r” for betaine and “GLYBt4(2)r” for GABA] need to be corrected for the requirement of both sodium and chloride ions. Therefore, the transport module contains the improved reactions for the ATB^0,+^ and BGT-1 transporters.

### Transport reaction module

The transport module was assembled according to the established reconstruction protocol (Thiele and Palsson, 2010a) using rBioNet as a reconstruction tool (Thorleifsson and Thiele, 2011). The functionality of reactions in the module, in conjunction with Recon 2, was subsequently tested. All of the discussed modifications and additions are provided through a transport module, which comprises of 71 metabolites, 70 reactions, and 41 genes (including 19 newly added genes). These additional transport reactions are for amino acids (27 reactions), lipids (16 reactions), nucleosides (6 reactions), vitamins and minerals (8 reactions), hormones (6 reactions), and others (7 reactions). In addition, 24 Recon 2 reactions need to be updated with respect to their gene-protein-reaction associations provided in Supplemental Table S2. Details of the transport module can be found in Supplemental Table S2 and also at http://humanmetabolism.org. Overall, the transport module summarizes in a computer-readable, structured manner all transport systems, and their corresponding reactions, that we discovered to be missing from Recon 2 (Figure 1H). This module is thus an extension to Recon 2, which can be added to the existing reconstruction if desired.

### Transport proteins associated with diseases

Transporters fulfill a broad range of functions, which go far beyond the sole movement of metabolites. In our discussion on the transport of distinct metabolite classes, many of these functions have been mentioned. Targeting specific transport proteins to combat disease conditions, such as cholestasis (Wagner and Trauner, 2005), neurodegenerative disorders (Hinoi et al., 2005), cystic fibrosis (Amaral and Kunzelmann, 2007), cancer (Lo et al., 2008; Ganapathy et al., 2009), cerebral ischemia (Kimelberg, 2005), diabetes, and secretory diarrhea (Wright et al., 2007), have gained considerable attention in recent years. Herein, we will focus on the discussion of transport proteins in disease groups concerning IEMs and cancer.

#### Transport proteins associated with metabolic diseases

Metabolic disorders are associated with disrupted cellular metabolism. IEMs are hereditary metabolic disorders, caused by specific mutations in genes encoding metabolic enzymes/transporters. We have recently mapped more than 200 IEMs onto the two human GENREs (Sahoo et al., 2012; Thiele et al., 2013). Of these, 14% (Table 4) were caused by faulty transport systems, including 15 IEM-causative genes associated with ABC transporters and 22 IEM-causative genes associated with SLC transporters. One good example for an IEM caused by a faulty transport system is lysinuric protein intolerance (OMIM: 222700). This disorder is caused by mutations in the gene encoding the y^+^ LAT1 amino acid transport system (*SLC7A7*, GeneID: 9056). Although rare (mostly observed in the Finish and Japanese populations, with incidence of 1:60,000 live births), this IEM has a clinical picture of recurrent diarrhea, vomiting, and in the long-term affects the immune system, skeletal system, and pulmonary and renal function, which can even lead to the death if left untreated. Specific dietary recommendations include protein restriction and citrulline and lysine supplementation. Recon 1 captured 22 of the 45 plasma membrane transport protein-associated IEMs genes. Recon 2 captured three additional diseases/genes. The remaining 20 transport protein-associated IEMs could not be mapped onto Recon 2 due to missing genes (see Table 4 for details). The IEMs for the ABC class of transporters account for 33% of these missing IEMs. The non-inclusion of the ABC transport proteins into human GENREs is because they have been shown to transport mainly medically important drugs and their derivatives (e.g., *ABCB1*, GeneID: 5243, *ABCG2*, GeneID: 9429) or that insufficient information on the preferred substrates is available (e.g., for *ABCF3*, GeneID: 55324). However, another major proportion of the missed IEMs/genes concerns fat-soluble vitamins and lipids (e.g., SRB-I, Niemann-Pick C1-like protein 1), for which transport mechanisms have only been partially resolved (see the relevant sections above). Therefore, we would like to emphasize the need to have sufficient information regarding the preferred substrates, associated cofactors/ions, substrate:ion stoichiometry, transport kinetics, and sub-cellular localization of transport proteins for building a high quality reconstruction of the transport reactions/pathways.

**Table 4:**
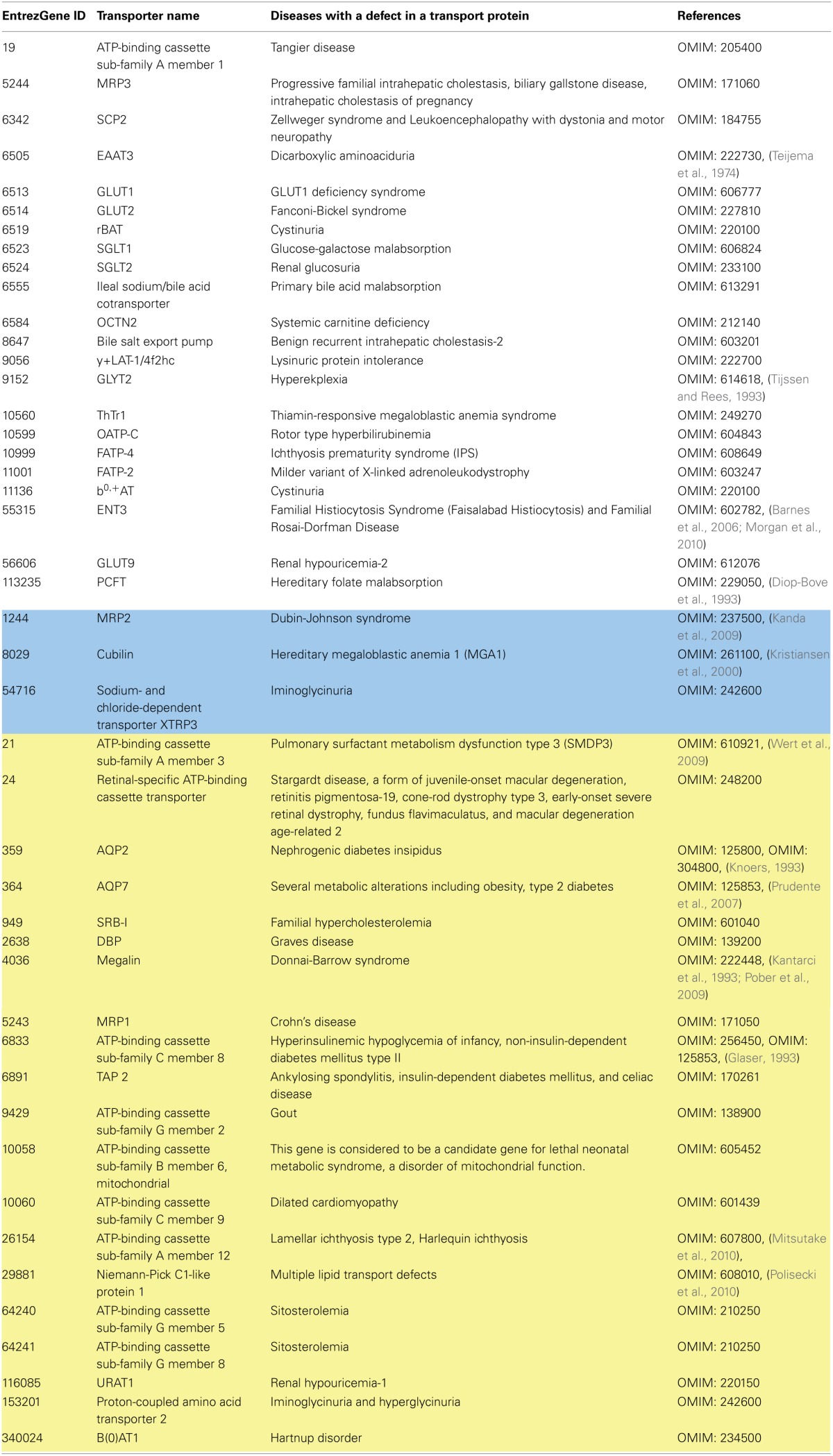
**Metabolic diseases associated with transport proteins**.

*The color coding is the same as in Table 1*.

#### Transport proteins associated with cancer

Cancer cells reprogram metabolic pathways to support their increased need for energy and biosynthetic precursors (Cairns et al., 2011). The metabolic characteristics of cancer cells are the high uptake of glucose, aerobic glycolysis, secretion of lactate (Warburg effect), and a high rate of glutaminolysis to compensate for the efflux of TCA cycle intermediates into biosynthetic pathways (Feron, 2009). Alternations in metabolite uptake (e.g., amino acids and glucose) and secretion through specific sets of transporters constitute key factors for how these continuously proliferating cells meet their metabolic demands (Ganapathy et al., 2009). As discussed above, redundancy and overlapping substrate specificity exist within and between transporter families. Cancer cells have to operate sets of transporters that best nourish their metabolic dependencies. In fact, the distinctive transporter expression between cancerous and normal cells could provide good opportunities for targeted treatment (Ganapathy et al., 2009). The contribution of transporters of the metabolite classes in cancer discussed above has been reviewed elsewhere (Fuchs and Bode, 2005; Verkman et al., 2008; Ganapathy et al., 2009; Calvo et al., 2010; Fletcher et al., 2010) and is summarized in Table 5.

**Table 5:**
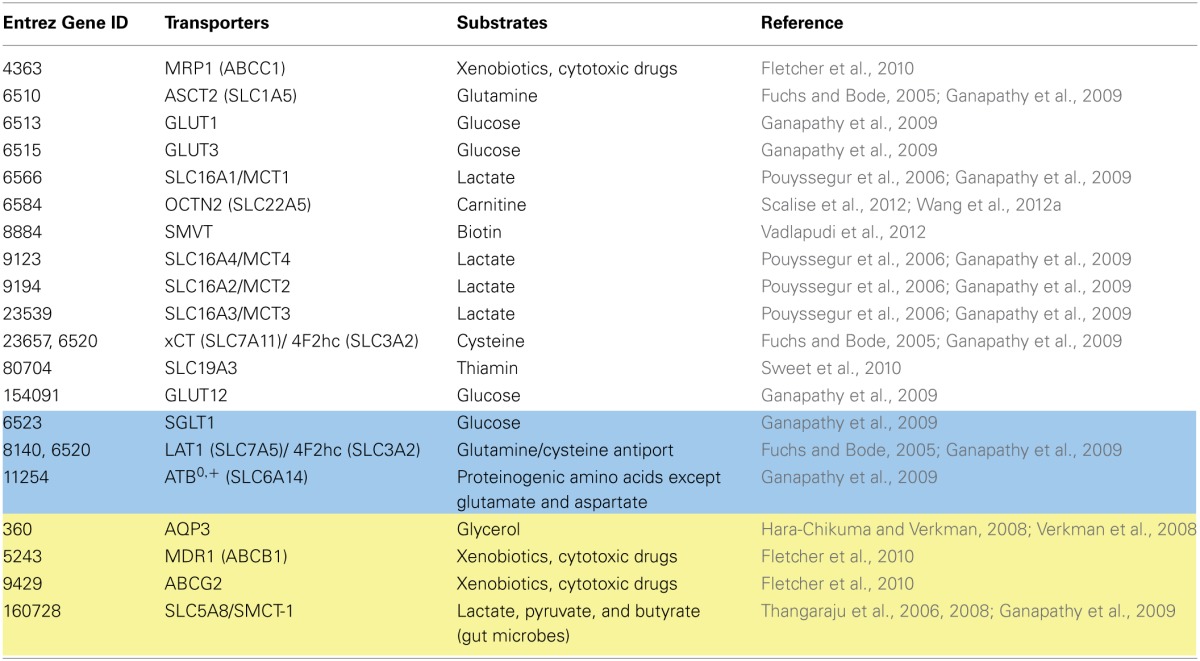
**Metabolite transporters relevant to cancer and their current coverage in Recon 2**.

*The color coding is the same as in Table 1*.

Coverage and accurate representation of transport systems are essential to perform valuable simulations using COBRA. Recon 1 has been used for the generation and analysis of cancer-specific metabolic models (Folger et al., 2011; Frezza et al., 2011; Jerby et al., 2012; Wang et al., 2012b) and has been recently summarized (Jerby and Ruppin, 2012; Hernández Patiño et al., 2013). Of the 20 extracellular transporters (Table 5) that play a role in cancer metabolic reprogramming and proliferation, 13 transporters are correctly represented in Recon 2 (Table 5), three need to be modified, and four are still missing or require further curation. This section discusses the cancer relevant transporters currently missing or requiring revision (Table 5).

The pyruvate to lactate conversion is necessary to sustain a high glycolytic flux (Feron, 2009). The accumulation of lactate and a decreasing pyruvate level put cell survival at risk due to increasing acidification of the cytoplasm. Cancer cells counteract the decrease in intracellular pH by specific ion transport (i.e., bicarbonate and protons) and lactate export via lactate/H^+^ symport, which is mediated by one of the four MTC transporters (*SLC16A1*, GeneID: 6566; *SLC16A7* GeneID: 9194; *SLC16A8* GeneID: 23539; *SLC16A4* GeneID: 9122). The high affinity lactate transporter SMCT1 (*SLC5A8*, GeneID: 160728) favors the import of lactate (Gopal et al., 2004) and is suppressed in a number of cancer cell types, as summarized in (Ganapathy et al., 2009). For example, *SLC5A8* is silenced by methylation in human astrocytomas and oligodendrogliomas (Hong et al., 2005) and in primary colon cancers and colon cancer cell lines (Li et al., 2003). In addition to its transporter function, the SLC5A8 protein has a demonstrated role in tumor suppression through the active import of endogenous inhibitors of histone acetylases (HDACs) [i.e., butyrate, which originates from gut microbes, and pyruvate (Thangaraju et al., 2006, 2008)]. Recently, SLC5A8 was shown to counteract tumor progression independent from its transport function. Instead, SLC5A8 acts through an unknown mechanism involving a decrease in the anti-apoptotic protein survivin (Coothankandaswamy et al., 2013). Recon 2 includes passive iodide transport via SLC5A8 and the Na^+^-coupled transport of lactate, pyruvate, and the short-chain fatty acids acetate, propionate, and butyrate (Miyauchi et al., 2004) (Table 5). Hence, these data were added in the transport module (Supplemental Table S2). SLC5A8 was not included in Recon 2, most likely because this protein has been mainly discussed in the context of cancer.

ABC transporters mediate the efflux of cytotoxic drugs, causing multidrug resistance (MDR) and chemotherapy failure (Fletcher et al., 2010; Falasca and Linton, 2012). Two of the four major drug transporters, MDR1 (*ABCB1*, GeneID: 5243) and ABCG2 (*ABCG2*, GeneID: 9429), are missing in Recon 2 (see also the IEMs section). Both are known to be overexpressed in different cancer types (Fletcher et al., 2010). A subpopulation of cancer cells with enriched stem cell activity, so called side populations (SPs), have been extracted from six human lung cancer cell lines (H460, H23, HTB-58, A549, H441, and H2170). When tested for an elevation in ABC transporter expression, all of the SPs displayed a significantly higher mRNA expression for ABCG2 compared to their non-SP counterparts (Ho et al., 2007). Four SPs also showed a significantly higher expression for MDR1 transporters. All six showed resistance to exposure to different chemotherapeutic drugs. The survival of such cells with stem cell activity upon drug treatment could be connected to a relapse *in vivo* (Ho et al., 2007), and ABC transporter expression might be an indicators for this cancer cell phenotype.

Strong expression of aquaporins has been observed in various tumors, especially aggressive tumors (Verkman et al., 2008). Some aquaporins are exclusively expressed in malignant tissue (Verkman et al., 2008). The aquaglyceroporin aquaporin-3, AQP3 (*AQP3*, GeneID: 360), which also transports glycerin in addition to water, is expressed in normal epidermis and overexpressed in basal cell carcinoma and human skin squamous cell carcinomas (Hara-Chikuma and Verkman, 2008). AQP3-facilitated glycerol transport was found to determine cellular ATP levels and therefore be important for hyperproliferation and tumor cell proliferation in epidermal mice cells (Hara-Chikuma and Verkman, 2008). Correspondingly, the resistance of AQP3 null-mice toward skin tumors might arise through reduced tumor cell glycerol metabolism and ATP generation (Hara-Chikuma and Verkman, 2008). This property renders AQP3 inhibition a possible target for the prevention and treatment of skin, and possibly other cancers associated with aquaglyceroporin overexpression (Hara-Chikuma and Verkman, 2008). AQP3 is currently missing in Recon 2 and covered in the transport module.

Although many of the transporters associated with cancer are present in Recon 2 (Table 5), important mediators of intra- and extracellular pH, drug resistance, and proliferative energy metabolism are still missing.

## Conclusion

A great deal of work in the field of constraint-based modeling has focused on the generation of highly curated GENREs and their usage for the generation of tissue-specific metabolic models for biomedical applications.

Transporters not only maintain the connectivity of metabolites across different cell types but also determine the uptake and secretion profile of individual cells. The metabolite exchanges of individual cell types with the corresponding extracellular compartment are inevitably connected to their internal biochemical pathways and cell functions. The inclusion of the cell type-specific transporters is important for enabling the use of the human metabolic reconstruction as a template for the generation of more accurate and physiologically relevant cell type-specific sub-networks and ultimately function-representative models. Moreover, information regarding distinct transporter function at different locations, as is the case for polarized cells, is crucial for such an effort and has thus been noted throughout this review. We identified numerous gaps through our literature review, many of which could be filled and are provided in the accompanying transport module. However, some knowledge gaps still remain because the responsible transporter or transport mechanism is unknown. Such a knowledge update for the GENRE needs to be performed periodically because of the important implications on their predictive potential (Thiele and Palsson, 2010b) and thus biomedical applications.

### Conflict of interest statement

The authors declare that the research was conducted in the absence of any commercial or financial relationships that could be construed as a potential conflict of interest.
